# Evaluation of Saliva Collection and DNA Extraction Methods for Practical Application of Salivary Human Herpesvirus 6 and 7 Assays

**DOI:** 10.3390/v17030411

**Published:** 2025-03-14

**Authors:** Shinsuke Tamai, Ryota Sone, Koichi Watanabe, Kazuhiro Shimizu

**Affiliations:** 1 Department of Sports Sciences, Japan Institute of Sports Sciences, Kita-ku 115-0056, Tokyo, Japan; kazuhiro.shimizu@jpnsport.go.jp; 2 Department of Clinical Medicine, Institute of Medicine, University of Tsukuba, Tsukuba 305-8577, Ibaraki, Japan; 3 Faculty of International Agriculture and Food Studies, Tokyo University of Agriculture, Setagaya-ku 156-8502, Tokyo, Japan; rs207923@nodai.ac.jp; 4 Institute of Health and Sport Sciences, University of Tsukuba, Tsukuba 305-8574, Ibaraki, Japan; watanabe.koichi.ga@u.tsukuba.ac.jp

**Keywords:** human herpesvirus 6 and 7, saliva collection, nucleic acid extraction, PCR, viral testing, practical application

## Abstract

Salivary human herpesvirus 6 and/or 7 (HHV-6/7) have recently attracted attention as microbiological markers of physiological fatigue in laborers and athletes. However, the accuracy and efficiency of the HHV-6/7 assays can be improved for practical application. We conducted three experiments to identify suitable saliva collection and DNA extraction methods for practical salivary HHV-6/7 assays. The main experiment compared the data, including template DNA or HHV-6/7 concentrations, among three saliva collection methods (cotton, synthetic, and no swabs) and two DNA extraction methods (magnetic bead-based and silica column-based). It showed that using swabs had adverse effects: lower template DNA concentration, lower HHV-6/7 detection rates, higher coefficient of variation values, and lower concentrations. Moreover, magnetic bead-based methods resulted in higher HHV-6/7 detection rates and lower coefficient of variation values. Sub-experiment 1 examined practical saliva collection methods and demonstrated that the stimulated spitting method could collect saliva in a shorter time with lower subjective stress than the unstimulated spitting and stimulated swabbing methods. Sub-experiment 2 investigated diurnal variation in salivary HHV-6/7 levels but did not show diurnal variation. These findings suggest that (1) the combination of stimulated spitting saliva collection and magnetic bead-based DNA extraction is most suitable for practical salivary HHV-6/7 assays, and (2) saliva collection can be conducted whenever needed.

## 1. Introduction

Salivary human herpesvirus 6 and/or 7 (HHV-6/7) have attracted attention as microbiological markers of physiological fatigue [1]. These viruses establish latent infection at a rate of more than 90% in the general adult population [2] and are highly detectable in the saliva of healthy individuals [3,4,5]; thus, they can be widely used as salivary biomarkers. After establishing a latent infection, these viruses are reactivated by endoplasmic reticulum stress, which may be involved in the mechanisms of physiological fatigue and physical adaptation in humans [6,7]. Several studies have reported the usability of salivary HHV-6/7 in the assessment of fatigue in laborers and athletes [1,8,9,10]. Moreover, recent studies have revealed that salivary HHV-6/7 levels are associated with the development of depression and myalgic encephalomyelitis/chronic fatigue syndrome [11,12]. Accordingly, salivary HHV-6/7 are expected to be useful for the daily assessment of physiological fatigue and the early detection of pathological fatigue in the clinical practice of occupational and sports medicine.

However, the following issues must be considered for practical application: (1) how to obtain accurate results efficiently, (2) how to implement the measurement in a manner that is less burdensome for the examinees, and (3) when the measurement should be performed.

For the first issue, the key to realizing a highly accurate salivary HHV-6/7 assay using quantitative polymerase chain reaction (qPCR) is to prepare a sufficient high-quality DNA template by selecting suitable saliva collection and DNA extraction methods. Saliva collection includes several methods [13], the most commonly used of which is spitting or swabbing. Although the swabbing method is sometimes used for viral testing, we suspect that the use of swabs may increase the false-negative rate because nucleic acids are adsorbed onto the swab. Several studies have shown that using swabs affects the concentration of salivary proteins or nucleic acids [14,15], and thus, the preferred collection method for salivary HHV-6/7 assays should be examined. Meanwhile, major DNA extraction methods include the phenol-chloroform, silica column-based (SC), and magnetic bead-based (MB) methods. Traditionally, phenol-chloroform extraction has been used, but it is considered unsuitable for practical application because it requires considerable time and effort and is inferior to SC extraction in terms of purity [16]. MB extraction has recently been developed to be more efficient through automation [17]; however, it is not known whether the SC or MB method is preferable for salivary HHV-6/7 assays.

For the second issue, the choice of the stimulated or unstimulated saliva collection method is the main point of discussion. Many studies have used the unstimulated method; however, it is expected to be more burdensome and waste more collection time for individuals with lower salivary secretions. Hence, it is necessary to select a method that reduces the time required for saliva collection while maintaining a low-stress level. However, no studies have examined subjective stress levels during saliva collection.

For the third issue, many salivary biomarkers (e.g., cortisol or testosterone) show diurnal variations [18]; thus, it is necessary to standardize the sampling time for the longitudinal measurement of intra-individual stress. However, diurnal variations in salivary HHV-6/7 levels remain unclear.

Therefore, three experiments were conducted to address each of these issues. Main experiment—Comparison of saliva collection and DNA extraction methods: We aimed to determine the effects of the saliva collection methods (with or without swabs and different swab materials) and DNA extraction methods on template DNA concentrations, HHV-6/7 detection rates, coefficient of variation (CV) values, and concentrations. We hypothesized that using swabs would negatively affect these items. Sub-experiment 1—Examination of practical saliva collection: The objective of this sub-experiment was to examine the effects of different saliva collection methods on examinees’ stress levels. Because chewing increases saliva secretion, we hypothesized that chewing would reduce subjective stress and allow the collection of a sufficient amount of saliva in a short time. Sub-experiment 2—Investigation of diurnal variation in salivary HHV-6/7 levels: We aimed to determine diurnal variations in salivary HHV-6/7 levels. We hypothesized that salivary HHV-6/7 levels would not exhibit diurnal variation because they are not physiological markers synthesized in the human body.

## 2. Materials and Methods

### 2.1. Ethical Approval

This study was approved by the ethics committee of the Japan Institute of Sports Sciences (2022-025). In accordance with the Declaration of Helsinki, all participants were informed in writing and verbally of the purpose, methods, and possible risks of the study. Written informed consent was obtained from all the participants. None of the participants complained of any discomfort or adverse physical condition during the study period.

### 2.2. Main Experiment—Comparison of Saliva Collection and DNA Extraction Methods

#### 2.2.1. Participants

We recruited participants by displaying posters in the Kanto region of Japan describing the purpose, method, and potential risks of the study, and a total of 30 healthy adults participated in this study (Table 1). The sample size was determined by referring to previous studies [16,19]. The participants gathered in a room at the University of Tsukuba on a convenient date between January and February 2023. They were instructed to refrain from consuming alcohol and caffeine within 12 h, eating within 1 h, and brushing their teeth with dental paste within 30 min before saliva collection.

#### 2.2.2. Saliva Collection

**Figure 1:**
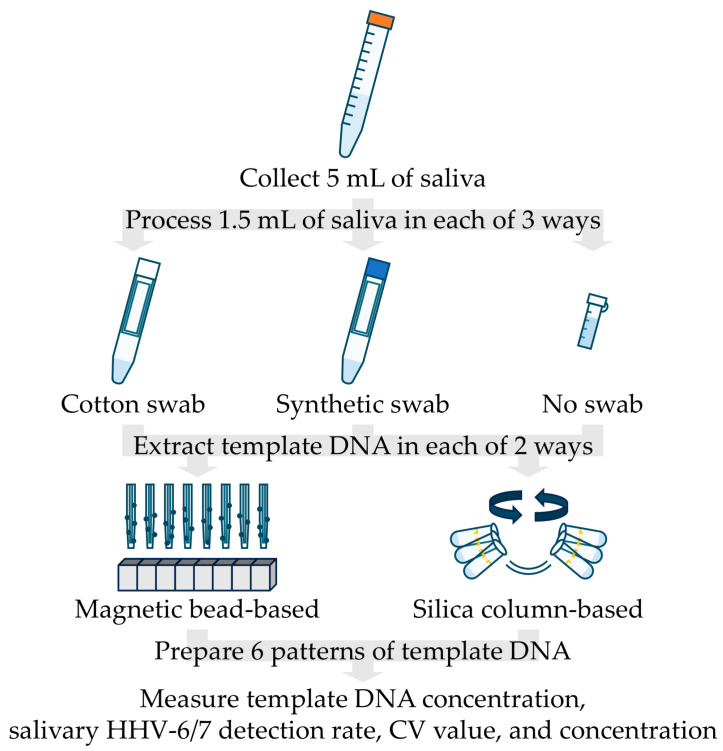
Protocol of the main experiment. HHV: human herpesvirus; CV: coefficient of variation.

An outline of this protocol is shown in Figure 1. On the day of sampling, approximately 5 mL of saliva was collected using a sterile straw and a 15 mL tube, according to the following procedure. The participants sat for 5 min and rinsed their oral cavities with distilled water three times for 30 s each. After 5 min, they swallowed the saliva stored in the oral cavity and chewed paraffin gum for saliva testing (Salivar Gum-α, Tokyo Shizaisha, Tokyo, Japan) once per second and spat their saliva into the 15 mL tube. Immediately after collecting saliva, the samples were stored at −80 °C until the day of the laboratory experiment. In the laboratory experiment, 1.5 mL of each saliva sample was processed under three conditions: cotton (Cot), synthetic (Syn), or no swabs (Non). After centrifuging the Cot and Syn samples at 4 °C and 3500 rpm for 15 min, these three conditions of saliva were dispensed into each 2 mL tube, and the collection volume was measured.

#### 2.2.3. DNA Extraction

DNA was extracted from saliva samples using either the MB or SC method. The MB method was performed using the Maxwell^®^ RSC Stabilized Saliva DNA Kit and Maxwell^®^ RSC Instrument (Promega, Madison, WI, USA) according to the manufacturer’s instructions. To 1000 µL of saliva sample, 30 µL of Proteinase K (Promega) was added, and the solution was incubated at 56 °C for 1 h on a heat block. DNA was extracted from 1000 µL of processed saliva and stored in 50 µL of elution buffer. The SC method was performed using the QIAamp MinElute Virus Spin Kit (Qiagen, Hilden, Germany) according to the manufacturer’s instructions. DNA was extracted from 200 µL of saliva and stored in 25 µL of elution buffer (the enrichment rates differed between the MB and SC methods to compare the maximum performance of each extraction method, which is in line with the objective of examining the practical aspects of this study). After extraction, the concentration and purity of DNA were determined using a NanoDrop™ Lite Spectrophotometer (Thermo Fisher Scientific, Waltham, MA, USA). Samples extracted using the SC method were found in preliminary experiments to have a minimum concentration of 148.2 ng/µL (*n* = 5), even when distilled water was used as the sample, due to the addition of carrier RNA. The template DNA concentration for each sample was normalized by subtracting this value.

#### 2.2.4. HHV-6/7 Assays

HHV-6/7 DNA in each sample was quantified using the TaqMan qPCR assay on a QuantStudio^®^ 3 Real-Time PCR System (Applied Biosystems, Foster City, CA, USA) as described previously, with some modifications [10]. qPCR was performed in a total volume of 10 µL containing 5 µL of premix, TaqPath qPCR Master Mix, CG (Applied Biosystems), 0.04 µL of PCR forward primer (50 µM), 0.04 µL of PCR reverse primer (50 µM), 0.25 µL of TaqMan probe (10 µM), 3 µL of DNA template, and 1.67 µL of distilled water. The primer sequences were as follows: HHV-6 forward primer, 5′-CAA AGC CAA ATT ATC CAG AGC G-3′; HHV-6 reverse primer 5′-CGC TAG GTT GAG AAT GAT CGA-3′, HHV-6 probe, 5′-FAM-CAC CAG ACG TCA CAC CCG AAG GAA T-MGB-3′; HHV-7 forward primer, 5′-ATG TAC CAA TAC GGT CCC ACT TG-3′; HHV-7 reverse primer, 5′-AGA GCT TGC GTT GTG CAT GTT-3′, HHV-7 probe, 5′- FAM-CAC GGC AAT AAC TCT AG-MGB-3′. The cycle conditions were 95 °C for 20 s, followed by 45 cycles of 95 °C for 3 s and 58 °C (for HHV-6) or 60 °C (for HHV-7) for 30 s. All samples were assayed in triplicate, and the median of the three values was used as the representative value because some low-concentration samples often show large deviations; using the median value helps to avoid artificial elimination. Salivary HHV-6/7 concentrations are expressed as log_10_ transformed copies/mL. The CV values were calculated by dividing the mean by the standard deviation of the three values; a lower CV value indicates greater accuracy.

#### 2.2.5. Statistical Analysis

All data are expressed as mean ± standard error of the mean (SE). All statistical analyses were performed using SPSS version 26 (IBM, Armonk, NY, USA). The Friedman test was used to examine the differences in the volume of saliva collected after processing the three conditions. Two-way analysis of variance (ANOVA) was used to examine the interaction between saliva collection and DNA extraction methods and their respective main effects, and the *p* values, *F* values, partial eta squared (η*_p_*^2^) values, and power values were calculated. When a significant interaction was found, the simple main effect was examined using the Bonferroni post hoc test. Statistical significance was set at *p* < 0.05.

### 2.3. Sub-Experiment 1—Examination of Practical Saliva Collection

#### 2.3.1. Participants

The participants were the same as those in the main experiment.

#### 2.3.2. Saliva Collection

After rinsing the oral cavity with distilled water three times for 30 s each and resting for 5 min, the following saliva collection methods were used: (1) unstimulated spitting (Unsti-Spt), (2) stimulated spitting (Sti-Spt), and (3) stimulated swabbing (Sti-Swb). For methods 1 and 2, a Saliva Collection Aid and Cryovial (Salimetrics, State College, PA, USA) set was used, and the time taken to collect 1 mL was measured. In methods 2 and 3, stimulation was performed by chewing once per second for 1 min using paraffin gum or cotton swabs, respectively. After collecting the saliva, the amount secreted per minute (mL/min) was determined.

#### 2.3.3. Questionnaire

The participants ranked the subjective stress levels of the three saliva collection methods.

#### 2.3.4. Statistical Analysis

All data are expressed as mean ± SE. Statistical analyses were performed using SPSS version 26 (IBM). The Friedman test was used to compare the amounts of saliva secreted. The chi-square test was used to rank the subjective stress levels according to the saliva collection methods. Statistical significance was set at *p* < 0.05.

### 2.4. Sub-Experiment 2—Investigation of Diurnal Variation in Salivary HHV-6/7 Levels

#### 2.4.1. Participants

Ten healthy adults participated in this experiment (Table 2). The participants were instructed to refrain from consuming alcohol and caffeine within 12 h, eating within 1 h, and brushing their teeth with dental paste within 30 min before saliva collection.

#### 2.4.2. Saliva Collection

Saliva was collected five times every 3 h, according to the following schedule: 8:00–9:00 (after waking up, before breakfast), 11:00–12:00 (before lunch), 14:00–15:00 (after lunch and before snacking, if they had), 17:00–18:00 (before dinner), and 20:00–21:00 (after dinner and before bed). The stimulated spitting method was used (as it was considered the preferred method based on the results of the main experiment and sub-experiment 1). Participants were provided with water for oral rinsing, pre-weighed 2 mL tubes, and paraffin gum in advance and were asked to choose a date and time when they were free from stressful events, such as exams or work. The participants were provided with the following instructions for saliva collection:

1. Rinse with the provided distilled water three times for 30 s each.
2. Rest in a sitting position for 5 min.
3. Place the paraffin gum in your mouth (do not touch with your hands directly) and have the sterile straw and 2 mL tube ready.
4. After swallowing the saliva stored in the mouth, start the stopwatch, chew the gum once per second, and spit out the saliva into the 2 mL tube.
5. After approximately 1.5 mL of saliva has been collected, stop the stopwatch and write down the “time of collection” and “time required for collection” in the provided paper.
6. Store the 2 mL tube containing saliva at −20 °C (the freezer compartment of a typical household refrigerator).

At a later date, we weighed the 2 mL tube containing the saliva sample, subtracted the pre-weighed amount, and divided it by the time required for collection to determine the amount of saliva secreted per minute (mL/min).

#### 2.4.3. DNA Extraction

The MB method was used for DNA extraction (which seemed to be the preferred method based on the results of the main experiment).

#### 2.4.4. HHV-6/7 Assays

HHV-6/7 measurements were performed as described above. Salivary HHV-6/7 levels were calculated by multiplying the DNA copy number by the amount of secreted saliva and were expressed as log_10_ transformed copies/min.

#### 2.4.5. Statistical Analysis

All data are expressed as mean ± SE. The Friedman test was performed using the SPSS Statistics version 26 (IBM). Statistical significance was set at *p* < 0.05.

## 3. Results

### 3.1. Main Experiment—Comparison of Saliva Collection and DNA Extraction

#### 3.1.1. Volume of Collected Saliva

After processing saliva samples in the three conditions, the mean ± SE volumes of the collected saliva were as follows: Non, 1.50 ± 0.00 mL; Cot, 1.29 ± 0.01 mL; Syn, 1.43 ± 0.00 mL. The Friedman test showed a significant difference among all groups (*p* < 0.01).

#### 3.1.2. Template DNA Concentrations and Purity

**Figure 2:**
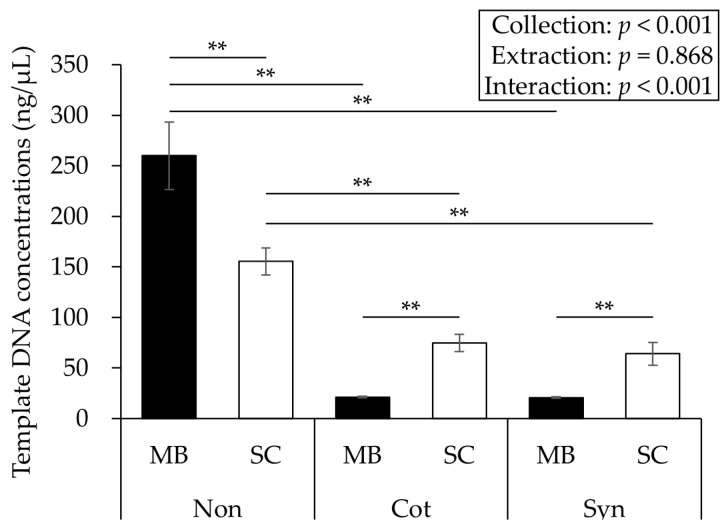
Comparison of the template DNA concentrations. Significant interaction was observed, and the Non-MB showed the highest concentration of the six conditions. Data are expressed as mean ± SE. The results of the post hoc test showed ** *p* < 0.01. Non: No swab; Cot: Cotton swab; Syn: Synthetic swab; MB: Magnetic bead-based; SC: Silica column-based.

Template DNA concentrations are shown in Figure 2. Two-way ANOVA revealed a significant interaction between the collection and extraction methods (*p* < 0.001, *F* = 13.228, η*_p_*^2^ = 0.486, power = 0.995). The means ± SE of the purity (A_260/280_) were as follows: Non-MB, 1.7 ± 0.0; Non-SC, 2.8 ± 0.0; Cot-MB, 1.4 ± 0.1; Cot-SC, 3.4 ± 0.0; Syn-MB, 1.3 ± 0.0; Syn-SC, 3.3 ± 0.0 (incidentally, a purity of approximately 1.8 is generally considered desirable, and cases much higher than 1.8 were likely to have been affected by the carrier RNA). Statistical analysis was not performed for purity because it was difficult to appropriately correct the SC-extracted data, which may have been affected by the carrier RNA.

#### 3.1.3. Salivary HHV-6/7 Assays

##### Detection Rates

The detection rates of HHV-6/7 are shown in Table 3. Although both detection rates should be 100% under all conditions, this was observed only for Non-MB.

##### CV Values

**Figure 3:**
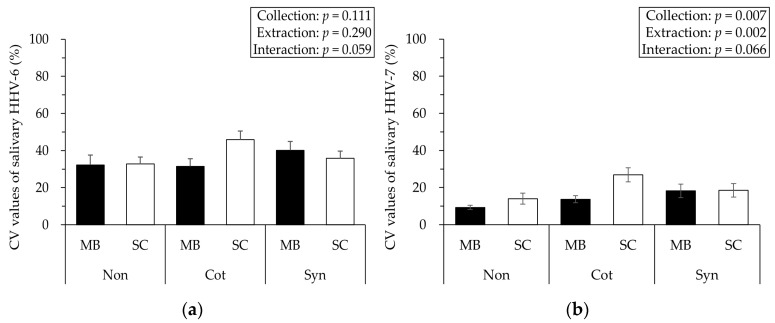
Comparison of the CV values of salivary HHV-6 (**a**) and HHV-7 (**b**). No interactions were observed for HHV-6/7. However, both CV values in the Non-MB were lower than the others, and the main effects of saliva collection and DNA extraction were observed for HHV-7. Data are expressed as mean ± SE. HHV: human herpesvirus; Non: No swab; Cot: Cotton swab; Syn: Synthetic swab; MB: Magnetic bead-based; SC: Silica column-based.

The CV values for HHV-6/7 are shown in Figure 3. Two-way ANOVA revealed no significant interaction for HHV-6 and HHV-7 (*p* = 0.059, *F* = 3.322, η*_p_*^2^ = 0.270, power = 0.554, and *p* = 0.066, *F* = 3.026, η*_p_*^2^ = 0.189, power = 0.536, respectively). There were no significant effects of collection or extraction for HHV-6 (*p* = 0.111, *F* = 2.487, η*_p_*^2^ = 0.216, power = 0.434, and *p* = 0.290, *F* = 1.184, η*_p_*^2^ = 0.059, power = 0.179, respectively); however, there were significant effects for HHV-7 (*p* = 0.007, *F* = 5.952, η*_p_*^2^ = 0.314, power = 0.838, and *p* = 0.002, *F* = 11.868, η*_p_*^2^ = 0.305, power = 0.913, respectively).

##### Concentrations

**Figure 4:**
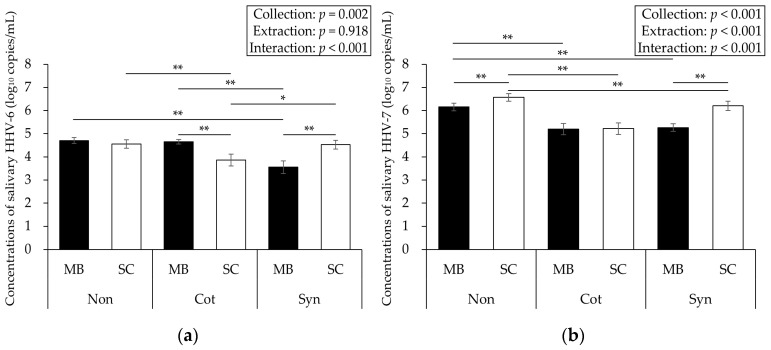
Comparison of the concentrations of salivary HHV-6 (**a**) and HHV-7 (**b**). Significant interactions were found for HHV-6/7; thus, these concentrations may be affected by the difference in the saliva collection and the DNA extraction methods, especially the use of cotton or synthetic swabs, which may result in lower concentrations. Data are expressed as mean ± SE. The results of the post hoc test showed * *p* < 0.05 or ** *p* < 0.01. HHV: human herpesvirus; Non: No swab; Cot: Cotton swab; Syn: Synthetic swab; MB: Magnetic bead-based; SC: Silica column-based.

The HHV-6/7 concentrations are shown in Figure 4. Two-way ANOVA showed significant interactions between saliva collection and DNA extraction for HHV-6/7 (*p* < 0.001, *F* = 16.586, η*_p_*^2^ = 0.542, power = 0.999, and *p* < 0.001, *F* = 71.609, η*_p_*^2^ = 0.836, power = 1.000, respectively).

### 3.2. Sub-Experiment 1—Examination of Practical Saliva Collection

#### 3.2.1. Saliva Secretion

The mean (SE) volumes of the saliva secretion were as follows: Unsti-Spt, 0.60 (0.06) mL; Sti-Spt, 1.57 (0.13) mL; Sti-Swb 1.38 (0.06) mL. The Friedman test showed a significant difference in saliva secretion volume between the different collection methods (*p* < 0.001), and a significant difference was observed between Unsti-Spt and Sti-Spt (*p* < 0.001), and Unsti-Spt and Sti-Swb (*p* < 0.001).

#### 3.2.2. Subjective Stress Level

Table 4 presents the subjective stress results for each saliva collection method. The chi-square test showed a significant difference (*p* < 0.001). While 22 participants indicated that the subjective stress of Unsti-Spt was high, none indicated that the subjective stress of Sti-Spt was high.

### 3.3. Sub-Experiment 2—Investigation of Diurnal Variation in Salivary HHV-6/7 Levels

**Figure 5:**
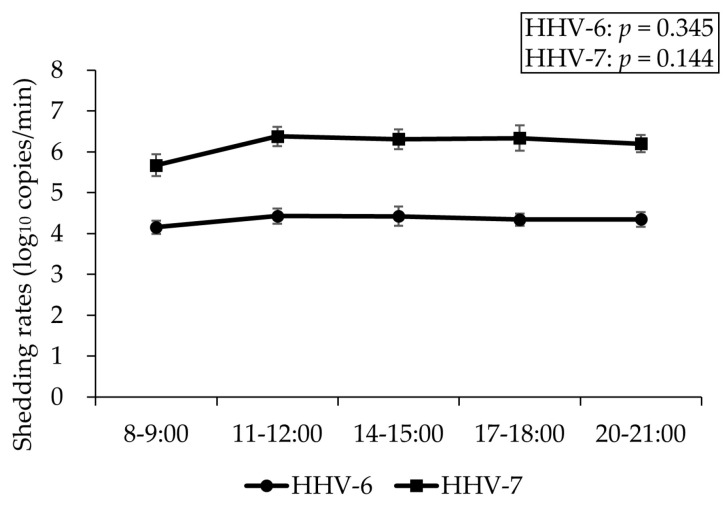
Diurnal variations of salivary HHV-6/7. Salivary HHV-6/7 levels did not show significant changes, suggesting no diurnal variations. Data are expressed as mean ± SE. HHV: human herpesvirus.

Diurnal variations in salivary HHV-6/7 levels are shown in Figure 5. Friedman test showed no significant changes in salivary HHV-6/7 levels (*p* = 0.345 and *p* = 0.144, respectively).

## 4. Discussion

Three experiments were conducted to establish effective saliva collection and DNA extraction methods for practical salivary HHV-6/7 assays. In the main experiment, we examined the effects of the presence or absence of swabs and different DNA extraction methods on template DNA concentration and qPCR data. Passing swabs decreased the amount of collected saliva and the template DNA concentration in both Cot and Syn samples. In addition, whether using the MB or SC methods, this can also increase the probability of false-negatives for HHV-6/7 and resulted in lower accuracy in qPCR data. In sub-experiment 1, the three saliva collections were compared, and Sti-Spt showed the highest saliva secretion volume and lowest subjective stress. In sub-experiment 2, we investigated the diurnal variation in salivary HHV-6/7 levels and found no significant variation. We integrated these results and discussed the aspects of saliva collection, DNA extraction, and timing of collection, considering their application in clinical practice.

The stimulated spitting method is considered the preferred method for saliva collection. This is because the use of swabs has the following negative effects: loss of saliva and DNA, increased false-negative rates and CV values, and decreased HHV-6/7 concentrations in qPCR data. Similarly, previous studies have examined the effect of different saliva collection methods on template DNA quantity and purity; the spitting method yielded the highest amount of extracted DNA [16]. This is likely because saliva and DNA are adsorbed by the swabs using the swabbing method. Additionally, the subjective stress level of Sti-Spt was the lowest, and increasing saliva secretion by chewing may help shorten the sample collection time. Furthermore, the swabbing method has a critical disadvantage in practical use, as the amount of saliva collected cannot be determined until the swab is centrifuged. Hence, there is a risk that the salivary HHV-6/7 assays cannot be performed if the amount of saliva sample does not reach the required volume after centrifuging. In contrast, in the spitting method, the amount of saliva sample is visible during collection; thus, saliva collection can be completed when a sufficient amount is reached, which can also help adjust the collection time, depending on individual differences. Regarding the choice between stimulated and unstimulated collection methods, the effect of chewing paraffin gum on salivary components is considered negligible [20,21]; hence, increased salivary secretion by chewing stimulation is considered to have a practical benefit. Moreover, saliva stabilization may not be essential when considering practical application (measurement time, load, and cost) because it has been reported that there is no difference in genomic DNA concentration without stabilization [22].

For DNA extraction, the MB method was preferable, although the difference in results was not as great as that observed with the saliva collection method. The first reason for this, the false-positives and false-negatives, must be avoided during clinical examinations. In the main experiment, the detection rates of salivary HHV-6/7 in the SC-extracted samples were lower than those in the MB-extracted samples. The second reason relates to measurement error in the assay. Our data showed that the CV value for HHV-7 in the MB method was lower than that in the SC method, which may be due to the quality of the DNA template. Lim et al. reported that the MB method produced the highest amount and purity of genomic DNA compared with SC and phenol-chloroform methods [16]. MB has also been reported to be superior to other methods for the extraction of bacterial DNA from saliva samples [23]. Although both the MB and SC methods showed better performance than several other extraction kits, the MB method has the advantage of a fully automated protocol over the examiner-intensive SC method [24]. The SC method is slightly inferior to the MB method in terms of the quality of the DNA template, which may be due to the adsorption of nucleic acids onto silica membranes [25]. However, although the MB method is relatively better, the extraction accuracy may vary among different models and specimens, even with the same MB method [19], and not all MB models are necessarily better than the SC method. A remaining question in the present study is why SC showed higher template DNA concentrations in Cot and Syn than in MB, whereas MB showed higher concentrations in Non. This might be because larger fragments, such as genomic DNA, are adsorbed on the swabs, whereas smaller fragments, such as carrier RNA, are relatively small and pass through it.

Furthermore, salivary HHV-6 and HHV-7 levels showed no diurnal variations. Although there are limited data, a previous study has reported that salivary HHV-4 (Epstein-Barr virus) did not show diurnal variations, which is consistent with our study [26]. The reason why salivary levels of HHVs did not show diurnal variation is unclear, but one possible explanation is that, unlike protein markers (e.g., cortisol or testosterone) synthesized in organisms, viruses are reactivated in response to stressors such as endoplasmic reticulum stress [6].

To summarize the appropriate methods for salivary HHV-6/7 assays for practical application, saliva samples should be collected by the spitting method using a commercial 15 mL sterile tube and sterile straw for saliva collection and chewing paraffin gum once per second until the required volume is obtained. In addition, the condition and duration of sample storage affects the quantified values [27]; thus, it is recommended that saliva samples are stored at −20 °C or lower after collection and to proceed to the DNA extraction and PCR steps as soon as possible. For DNA extraction, the MB method may be preferred for preparing DNA templates because it improves accuracy and efficiency. If such an automated instrument is difficult to use, it would be appropriate to extract DNA manually using the SC method. As for qPCR, salivary HHV-6 often shows higher CV value owing to its low concentration in saliva. Presently, it may be preferable to measure HHV-6 in triplicate and use the median value. Additionally, increasing the volume of the DNA template during qPCR from 3 to 4 µL is effective in reducing the measurement error for low-concentration samples. In the case of using salivary HHV-6/7 to assess physiological fatigue, saliva can be collected at any time for the assays because there is no diurnal variation in salivary HHV-6/7 levels (it is generally recommended that saliva samples are collected before breakfast.) For reference in further studies, we present the mean ± SE values of log_10_ transformed concentrations and shedding rates of HHV-6/7 measured by this method: concentration of HHV-6, 4.5 ± 0.1 copies/mL (male, 4.5 ± 0.2 copies/mL; female, 4.5 ± 0.2 copies/mL); concentration of HHV-7, 6.0 ± 0.2 copies/mL (male, 6.3 ± 0.2 copies/mL; female, 5.7 ± 0.2 copies/mL); shedding rates of HHV-6, 4.6 ± 0.1 copies/min (male, 4.7 ± 0.2 copies/min; female, 4.6 ± 0.2 copies/min); shedding rates of HHV-7, 6.1 ± 0.2 copies/min (male, 6.4 ± 0.2 copies/min; female, 5.8 ± 0.2 copies/min).

As a limitation for generalization, the saliva collection and DNA extraction methods presented in this study may also be applicable to other salivary component assays or possibly not all. In a previous study that investigated the effect of swabs on the values of protein markers, sample collection without swabs resulted in higher cortisol values, whereas collection with swabs resulted in higher secretory immunoglobulin A values [14]. Thus, different results are expected for different targets, and it is necessary to confirm which collection and extraction methods are best for measuring other targets. Specifically, in viral testing, although saliva is gradually gaining recognition as an alternative biofluid to nasopharyngeal or other samples [28], few studies have examined the effects of different saliva collection methods on viral testing. The swabbing method has been reported to be useful for detecting SARS-CoV-2 or Influenza virus in practical situations [29,30], but the stimulated spitting method has the potential to improve the accuracy of viral testing; thus, future studies should carefully consider this issue. Furthermore, the participants in the present study were recruited in a limited region, which may yield different results in other regions or populations.

## 5. Conclusions

The present study conducted three experiments to establish the practical salivary HHV-6/7 assays. Collectively, the results indicate that the stimulated spitting method is preferred for saliva collection because the use of swabs has negative effects, such as the loss of saliva and DNA, which leads to inhibit salivary HHV-6/7 assays. Additionally, the MB method is considered the preferred method for DNA extraction from the perspectives of increased template DNA concentrations, prevention of false-negatives, and reduced burden on examiners. Moreover, as no diurnal variation in salivary HHV-6/7 was observed, it is not necessary to standardize the sampling time for longitudinal observation. These findings may contribute to the practical application of salivary HHV-6/7 assays as well as the detection of other viruses in saliva.

## Figures and Tables

**Table 1:** Characteristics of the participants in the main experiment and sub-experiment 1.

Items	
Male–Female (*n*)	15:15
Age (years)	25.7 ± 2.6
Height (cm)	167.0 ± 11.4
Weight (kg)	64.2 ± 12.1
Average time of static labor ^1^ per day (h)	6.7 ± 3.1
Average time of dynamic labor ^2^ per day (h)	1.4 ± 1.8
Average time of sleeping per day (h)	7.2 ± 1.0

The data are expressed as mean ± SD. ^1^ Static labor refers to deskwork or classroom learning. ^2^ Dynamic labor refers to hard work or practical training.

**Table 2:** Characteristics of the participants in sub-experiment 2.

Items	
Male–Female (*n*)	5:5
Age (years)	27.5 ± 1.5
Height (cm)	166.5 ± 12.2
Weight (kg)	63.7 ± 9.4
Average time of static labor ^1^ per day (h)	9.4 ± 2.3
Average time of dynamic labor ^2^ per day (h)	0.1 ± 0.2
Average time of sleeping per day (h)	7.0 ± 0.9

The data are expressed as mean ± SD. ^1^ Static labor refers to deskwork or classroom learning. ^2^ Dynamic labor refers to hard work or practical training.

**Table 3:** Comparison of the detection rates of salivary HHV-6/7.

	Non		Cot		Syn	
	MB	SC	MB	SC	MB	SC
HHV-6	100% (30/30)	96.7% (29/30)	100% (30/30)	90.0% (27/30)	86.6% (26/30)	96.7% (29/30)
HHV-7	100% (30/30)	100% (30/30)	96.7% (29/30)	96.7% (29/30)	100% (30/30)	100% (30/30)

Data are presented as percentages (number of participants). HHV: human herpesvirus; Non: No swab; Cot: Cotton swab; Syn: Synthetic swab; MB: Magnetic bead-based; SC: Silica column-based.

**Table 4:** Comparison of the subjective stress levels.

Stress Level	Unsti-Spt	Sti-Spt	Sti-Swb
High	22 (5.7)	0 (−4.7)	8 (−0.9)
Middle	6 (−1.9)	15 (2.4)	9 (−0.5)
Low	2 (−3.8)	15 (2.4)	13 (1.4)

Data are presented as the number of participants (adjusted residuals). If the adjusted residual is >1.96 or <−1.96, it is considered statistically significant. Unsti-Spt: Unstimulated spitting method; Sti-Spt: Stimulated spitting method; Sti-Swb: Stimulated swabbing method.

## Data Availability

The data presented in this study are available on request from the corresponding author.
